# The Effect of Diet on Midgut and Resulting Changes in Infectiousness of AcMNPV Baculovirus in the Cabbage Looper, *Trichoplusia ni*

**DOI:** 10.3389/fphys.2018.01348

**Published:** 2018-10-04

**Authors:** Elizabeth Chen, Dennis Kolosov, Michael J. O'Donnell, Martin A. Erlandson, Jeremy N. McNeil, Cam Donly

**Affiliations:** ^1^London Research and Development Centre, Agriculture and Agri-Food Canada, London, ON, Canada; ^2^Department of Biology, University of Western Ontario, London, ON, Canada; ^3^Department of Biology, McMaster University, Hamilton, ON, Canada; ^4^Saskatoon Research and Development Centre, Agriculture and Agri-Food Canada, Saskatoon, SK, Canada

**Keywords:** cabbage looper, AcMNPV baculovirus, chitinase, chitin deacetylase, midgut transcriptome, pathogen resistance, peritrophic matrix, midgut pH

## Abstract

Insecticide resistance has been reported in many important agricultural pests, and alternative management methods are required. Baculoviruses qualify as an effective, yet environmentally benign, biocontrol agent but their efficacy against generalist herbivores may be influenced by diet. However, few studies have investigated the tritrophic interactions of plant, pest, and pathogen from both a gene expression and physiological perspective. Here we use microscopy and transcriptomics to examine how diet affects the structure of peritrophic matrix (PM) in *Trichoplusia ni* larvae and consequently their susceptibility to the baculovirus, AcMNPV. Larvae raised on potato leaves had lower transcript levels for chitinase and chitin deacetylase genes, and possessed a thicker and more multi-layered PM than those raised on cabbage or artificial diet, which could contribute to their significantly lower susceptibility to the baculovirus. The consequences of these changes underline the importance of considering dietary influences on pathogen susceptibility in pest management strategies.

## Introduction

Baculoviruses are entomopathogens with high host specificity but some, such as the Autographa californica multicapsid nucleopolyhedrovirus (AcMNPV), can infect a wide range of lepidopterans (Clem and Passarelli, 2013). Once ingested, the alkaline pH of the larval midgut dissolves the viral occlusion body (OB), releasing the occlusion-derived virions (ODVs) that rapidly pass through the midgut peritrophic matrix (PM) and infect the microvilli of columnar midgut epithelium cells (Granados and Lawler, 1981). However, the infection process can be affected by the larval food source (Duffey et al., 1995; Cory and Hoover, 2006) as this shapes the environment of the gut (Keating et al., 1990; Brodersen et al., 2012). Secondary metabolites (phenolics, flavonoids, and tannins) affect the palatability of plant tissue and influence caterpillar growth, which in turn affects larval behavior and plant-pest interactions (Sarfraz et al., 2011).

*Brassica* spp. synthesize glucosinolates, a significant class of natural defense compounds, which when ingested by insects are hydrolysed by myrosinase to produce isothiocyanates, oxazolidine thiones, epithionitroles, and nitrils (sometimes known as the “mustard oil bomb,” Grubb and Abel, 2006). These compounds are toxic repellents to some insects (Mithen et al., 2000; Lambrix et al., 2001; Talalay and Fahey, 2001; Agrawal and Kurashige, 2003) but others have evolved counter-adaptations including reduced plant cell disruption, rapid absorption of intact glucosinolates and rapid metabolic conversion of glucosinolates to harmless compounds (Winde and Wittstock, 2011). Although the functional basis of glucosinolate detoxification remains to be determined, there is transcriptomic evidence that glutathione S-transferases and cytochrome P450s are involved (Wadleigh and Yu, 1988; Whiteman et al., 2012; Kumar et al., 2013). *Solanum* spp. produce glycoalkaloids as natural defense compounds which inhibit acetylcholinesterase and butyrylcholinesterase, both of which catalyze the hydrolysis of the neurotransmitter acetylcholine, as well as interfering with calcium and sodium ion transport across cell membranes (Schwarz et al., 1995). In insect herbivores, the mechanisms of counter-adaptation are predominantly present in the fat body, which contains detoxification enzymes and ABC transporters (Yang et al., 2007).

Lepidopterans have both biochemical and physical gut attributes to counteract the challenges of a wide diet range. As one of the most alkaline environments in a natural system, a high midgut pH retains the nutritional quality of ingested plant proteins and offsets the toxicity of their host plant's defensive compounds (Woodham, 1983). Maintaining a pH > 8 prevents the antidigestive effects of tannin-protein-aggregates (Felton and Duffey, 1991). The physical peritrophic matrix (PM), which separates the gut content from the midgut wall, is another protective feature of the lepidopteran midgut. The PM consists of a proteinaceous matrix embedded in a chitin substructure that serves in the combined roles of digestion and absorption of nutrients, mechanical protection, toxin nullification, and pathogen restriction (Spence, 1991). However, while more toxic diets can induce thicker PM formation which affords greater protection from toxins and pathogens, there is a possible trade-off relating to digestive efficiency. For example, the growth rate of *Heliothis virescens* was reported to be lower when larvae were fed a more toxic diet that resulted in a thicker PM, although there was an additional effect as the larvae had a lower susceptibility to viral pathogens (Plymale et al., 2008).

*Trichoplusia ni* is a generalist herbivore capable of feeding on plants from many families, including Solanaceae and Brassicaceae. Its larval PM is typical of most lepidopterans, forming as a thin layer at the anterior end of the midgut and becoming progressively thicker as additional components are added to its structure by the midgut epithelium (Adang and Spence, 1981; Harper and Granados, 1999; Toprak et al., 2014). As it is thinnest and most porous at the anterior region this could help explain why the vast majority of AcMNPV infections in *T. ni* are initiated in the anterior midgut (Javed et al., 2016). The components contributing to PM architecture, synthesis, and function include structural (peritrophins, mucins, glycoproteins, lipases), microvesicle delivery (gelsolin, annexin), framework (chitin synthase, chitinase, chitin deacetylase), and hormonal effectors (ecdysone, juvenile hormone related) (Hegedus et al., 2016).

In the present study, we undertook experiments to confirm that variable diets could alter the susceptibility of *T. ni* larvae to viral infections (Cory and Hoover, 2006; Shikano et al., 2010) and to determine if larval diet affected the structure of the PM. In addition, using RNA-seq analysis, we investigated larval midgut gene expression to determine if the expression of various categories of genes whose products could influence PM structure differed with diet.

## Materials and methods

### Insect rearing, diet, and virus

All insects used were obtained from a *T. ni* culture reared on cabbage under a 16L:8D photoperiod at 25°C at Agriculture and Agri-Food Canada's (AAFC) London Research and Development Centre (LRDC). Golden Acre Cabbage (*Brassica oleraceae*) and Kennebec Potato (*Solanum tuberosum*) were grown in a greenhouse at 16L:8D photoperiod at 25°C while the McMorran wheat germ-based artificial diet (Adkisson et al., 1960) was purchased from Insect Production Services (Great Lakes Forestry Centre, Sault Ste. Marie, ON, Canada). Virus used for infections was derived from the AcMNPV bacmid bMON14272 backbone repaired with *gfp* under the control of the OpMNPV *ie1* promoter (Javed et al., 2016).

### Bioassays

**Figure 1:**
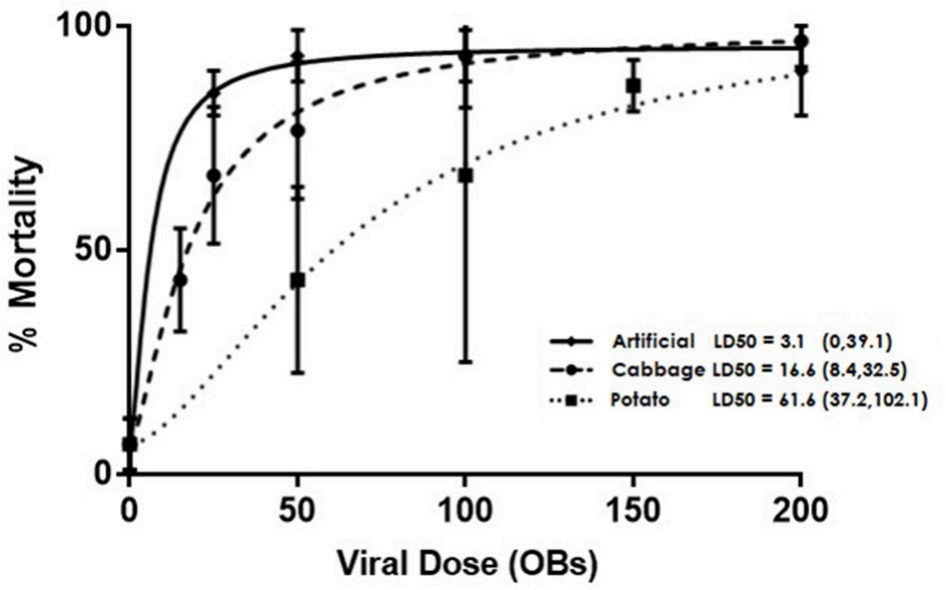
Dose dependent mortality of AcMNPV-treated 4th instar *T. ni* larvae that were raised on artificial diet, cabbage, or potato. Error bars depict 95% confidence intervals.

Fourth instar larvae were placed in 5.5 cm diameter petri dishes lined with moist filter paper and provided with either a 6 mm diameter potato or cabbage leaf disc (cut from the leaves of plants that were ~1 month old) or a 5 mm^3^ cube of artificial diet. The food samples were treated with viral doses calculated from preliminary bioassays: 15, 25, 50, 100, or 200 OBs on cabbage, 50, 100, 150, 250, or 400 OBs on potato, or 25, 50, 100, 200, or 350 OBs on artificial diet. The concentrations obtained using serial dilution were confirmed with hemocytometer counts. Controls for the three diet treatments were treated with 0.05% Triton X-100 in water, the solution used to suspend the OBs. In all cases, the virus was applied in a 2 μl droplet and on leaf disks the droplet was allowed to dry before being fed to insects. In the case of artificial diet, a square of parafilm was placed underneath the cube to ensure the solution was not absorbed by the filter paper. Once the treated food was completely consumed, untreated food was provided for 7 days post treatment or until the larvae pupated. There were three replicates of ten larvae per concentration per diet treatment and mortality was recorded daily. LD_50_ values were estimated and Fieller's method used to calculate subsequent 95% confidence intervals in the R add-on package *drc* (Cox, 1990; Ritz et al., 2015). Each continuous dose response curve was modeled based on the parameters of identical lower and upper limits with only binomial responses (alive or dead) and the results were compared pairwise within *drc* for identity based on slope and LD_50_ using a one-way ANOVA. GraphPad Prism software was used to generate Figure 1 showing the curves.

### Double-barrelled ion-selective microelectrode midgut pH measurements

Construction of double-barrelled ion-selective microelectrodes has been described in detail in Ianowski et al. (2002) and Ianowski and O'Donnell (2004) so the modified protocol used is described briefly. Double-barrelled “theta” glass microcapillaries were pulled to a fine point using a vertical micropipette puller PE-2 (Narashige, Japan). The pH-barrel was silanized to aid retention of the hydrophobic H^+^ ionophore cocktail, as described by Ianowski et al. (2002). The ion-selective barrel was then filled with a solution containing 100 mM sodium citrate and 100 mM NaCl (pH = 6.0). The reference barrel was filled with 150 mM KCl. Finally, the ion-selective barrel was tip-filled with Hydrogen ionophore I cocktail B. Each barrel was connected through chlorided silver wires to the inputs of a high-input impedance differential electrometer (HiZ-223, Warner Instruments, Hamden, CT) which was referenced to a bath electrode filled with 3M KCl in 4% agar. The pH microelectrodes were calibrated in TAPS-buffered solutions of pH 8, 9, and 10 with ionic strength approximating that of *T. ni* diet.

Ten 4th instar larvae from each of the three diets were pinned straight while submerged in saline solution. A longitudinal incision was made along the length of the caterpillar to expose the midgut so that a pH microelectrode could be inserted into the anterior lumen of the midgut and the pH recorded. All measurements were performed *in situ*, i.e., without removing the gut from the animal and completed within 5 min from the end of dissection. Microelectrode voltages were low-pass filtered at 2 Hz, digitized and recorded using a PowerLab data acquisition system (AD Instruments, Colorado Springs, CO) and analyzed using Chart software (Ad Instruments). pH values were calculated from microelectrode voltages which were stable to within 0.1 mV over 10 s. The pH data for the three diets were analyzed using a one-way ANOVA and assessed for significance using the Holm–Sidak method (Sidak, 1967; Holm, 1979).

### Scanning electron microscopy (SEM)

Larvae were reared on each of the three diets until the 4th instar (head capsule size 1.0–1.3 mm), at which time their midguts were dissected out, the food bolus removed, so that the PM could be cut lengthwise and immersed in Sorenson's phosphate buffer (pH 7.4) containing 2.5% glutaraldehyde as a primary fixative. After triple buffer rinsing, samples were fixed in 1% osmium tetroxide for 1 h, triple rinsed again, and then dehydrated in a graded ethanol series before being dried in a graded hexamethyldisilazane series (Shivley and Miller, 2009). Samples were mounted, gold sputtered, and observed with a Hitachi 3400-N VP-SEM. Three midgut samples per diet category were processed and the anterior, middle, and posterior regions of the resulting PMs from the larvae reared on differing diets were compared.

### Transmission electron microscopy (TEM)

The initial steps of TEM preparation were identical to those used for SEM, up to and including the fixation in 1% osmium tetroxide and secondary wash. Following that, TEM preparation included dehydration in a graded acetone series before infiltration and embedding in epon-araldite resin and finally baking at 60°C for 48 h. Coarse trimming was done using a razor blade and finer sectioning done on a Sorval Ultracut with a diamond knife. All sections were from the anterior region of the PM and netted on copper grids. Samples were post stained in the dark for 20 min with uranyl acetate, rinsed in five water droplets, stained for 2 min with lead citrate, and rinsed in three water droplets. Grid samples were examined and images taken using a Philips CM10 TEM 60 KV. The midguts from three larvae from each diet category were examined.

### RNA extraction, RNA-seq, and transcriptomic analysis

Midguts of 4th instar larvae from each diet category were dissected in Calpode's insect saline (pH = 7.2, 10.7 mM NaCl, 25.8 mM KCl, 90 mM glucose, 29 mM CaCl_2_, 20 mM MgCl_2_, and 5 mM HEPES), immediately suspended in RNAlater buffer (Ambion, Fisher Scientific) and stored at −20°C until RNA extraction. Total RNA was extracted from five pooled midguts using an RNeasy mini kit (Qiagen), and replicated three times for each diet treatment. The quality and quantity of RNA were assessed using a 2100 Bioanalyzer (Agilent). RNA samples were diluted to 300 ng/μL in DEPC-treated water. Strand-specific libraries were constructed and sequencing performed on the Illumina HiSeq 2000 platform using 100 bp single-end reads protocol at McGill University and Génome Québec Innovation Centre (Montreal, QC, Canada).

Messenger RNA-seq reads were mapped to a reference *T. ni* transcriptome containing 58,200 contigs that was previously assembled from 3 tissue-specific transcriptomes (Javed et al., 2017). Subsequent analysis and data handling were done using CLC Genomics Workbench version 10.0.1 (Qiagen Bioinformatics). Before mapping, library reads with <50 nucleotides were discarded. Gene expression tracks were then generated from RNA-seq analysis. Finally, all potential pairings of different diets were tested for differential expression. The output from DESeq analysis included normalized mean number of reads assigned to a contig, log_2_ fold change, and its statistical significance. A contig was considered differentially expressed if the absolute value of the log_2_ fold change was ≥3 and the adjusted *p*-value was ≤ 0.001 following Benjamini-Hochberg false discovery rate (FDR) correction (Benjamini and Yekutieli, 2001). Contigs corresponding to genes of interest were screened from all those found to be differentially expressed. Contig IDs without annotation were analyzed by BLASTN comparison with the NCBI database. The NCBI accessions and descriptions of matching entries using an E-value cut-off of 0.001 are reported in Supplementary Table 1.

## Results

### Diet based *T. ni* bioassay lethality curves

Larval diets had a significant effect on the susceptibility of *T. ni* larvae to AcMNPV baculovirus (Figure 1), with LD_50s_ of 3.1 OBs (95% CI: 0, 39.1); 16.6 OBs (95% CI: 8.4, 32.5); and 61.6 OBs (95% CI: 37.2, 102.1) for larvae reared on artificial diet, cabbage, and potato respectively. The LD_50s_ for larvae reared on artificial diet and cabbage did not differ significantly (*p* > 0.05, F-ratio = 2.76, *df* = 2) but both differed significantly from the LD_50_ value for larvae reared on potato (cabbage: *p* = 0.0019, F-ratio = 7.81, *df* = 2; artificial diet: *p* < 0.001, F-ratio = 14.16, *df* = 2).

### Measurements of anterior midgut pH

**Figure 2:**
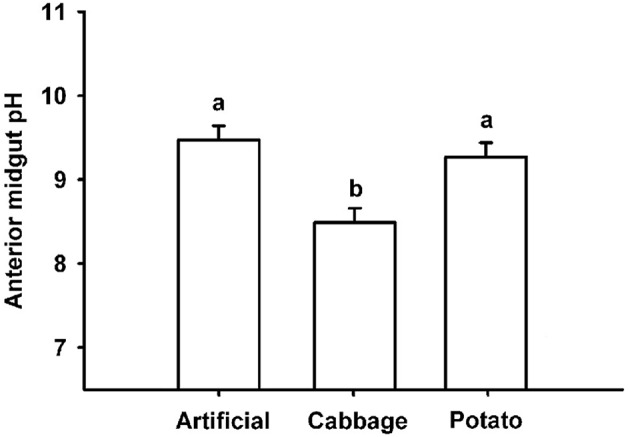
pH values in anterior midguts of 4th instar *T. ni* raised on artificial diet, cabbage, and potato. Different letters represent significant differences *p* < 0.001 using a one-way ANOVA coupled with a *post-hoc* multiple comparison Holm–Sidak test.

Diet had a significant effect on the pH in the anterior midgut of 4th instar *T. ni*, with larvae raised on cabbage having a significantly less alkaline pH (X = 8.44; SD ± 0.26) than those raised on artificial diet (X = 9.48; SD ± 0.53) or potato (X = 9.28; SD ± 0.52) (Figure 2). Statistically significant differences were determined using a one-way ANOVA coupled with a Holm-Sidak multiple comparison test (*p* < 0.001, F-ratio = 14.726). The degrees of freedom between groups = 2; the residual degrees of freedom = 27.

### SEM characterization of the PM

**Figure 3:**
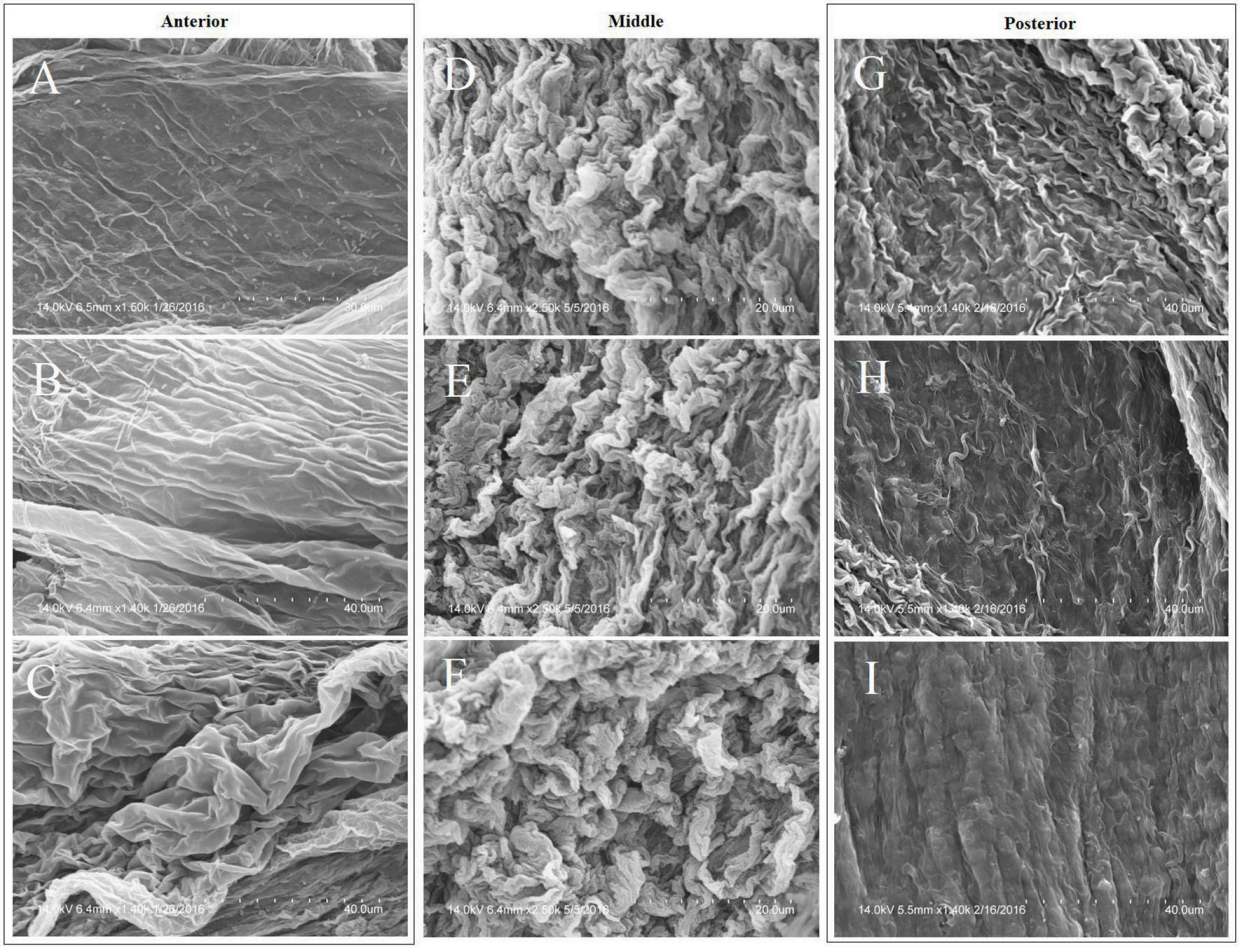
Morphological variation in the anterior, middle, and posterior sections of the PM of a cabbage-raised *T. ni* midgut per regional characteristics: left column = anterior; middle column = middle; right column = posterior. Magnification of images are: **(A)** = 1,500×; **(B)** = 1,400×; **(C)** = 1,400×; **(D–F)** = 2,500×; **(G–I)** = 1,400×. Images **(A–I)** are in spatial, sequential order along the length of the midgut.

The structural differences between the anterior, middle, and posterior sections of the midgut are presented in Figure 3. The anterior region has relatively shallow but extensive folds, transitioning into tight coils in the middle region and becoming degraded and sloughed at the posterior end.

While the anterior region of the PM can be morphologically differentiated from the middle and posterior sections, significant morphological differences between the anterior regions of larvae raised on the three diets were not observed.

### TEM characterization of the PM

**Figure 4:**
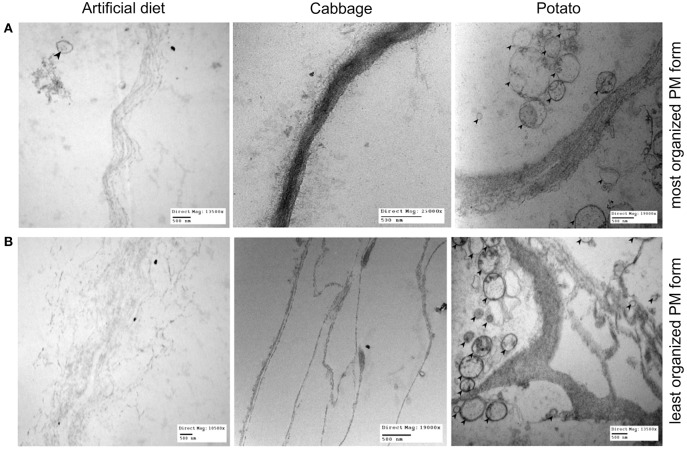
The range of PM morphology observed in 4th instar *T. ni* larvae raised on 3 different diets: artificial diet, cabbage, and potato. **(A)** Depicts an example of the most organized appearance of the PM for each diet and **(B)** depicts an example of the least organized. Note the bends/kinks present in the example of PM of potato-fed larvae at its least organized (bottom right image). Left column: larvae raised on artificial diet (top image magnification = 13,500×; bottom image magnification = 10,500×). Middle column: larvae raised on cabbage (top image magnification = 25,000×; bottom image magnification = 19,000×). Right column: larvae raised on potato (top image magnification = 10,500×; bottom image magnification = 13,500×). Bars = 500 nm. Black arrows point to microvesicles, which are present significantly more in potato-fed larval PMs regardless of the level of organization.

TEM imaging revealed significant morphological variability of the *T. ni* PM, both within and between diets, and the simplest morphologies are shown in Figure 4A. The PM of larvae reared on both artificial diet and cabbage is thin (≤500 nm in thickness) but with a more dense and uniform appearance in the cabbage treatment. In potato-raised larvae, the PM is usually thin (≤500 nm in thickness) but with its layers separated, thus spanning several microns.

By comparison, the most complex forms of PM (Figure 4B) have very different levels of organization, having thicker, less organized and more numerous layers than those shown in Figure 4A. Larvae reared on artificial diet vary from having a PM that exhibits separated laminae, spanning several microns, to one that is almost completely unraveled, with little structural integrity. In cabbage-raised larvae, the PM has divided laminae and further divided internal fibrils spanning several microns, but each layer is well defined. The structure of the PM in the potato treatment has bends and kinks of multiple fibrils and divided laminae, showing a much greater level of disorganization than in larvae from the other two diet treatments. This highly disorganized form of the PM in larvae reared on potato is the main form present and its prevalence over the simple form was greatest in this treatment group. Furthermore, regardless of form, the PM of larvae raised on potato also featured more numerous microvesicles (Figures 4A,B).

### mRNA sequenced read processing and quality control

The sequencing of 9 libraries constructed from larval midgut RNA extracted from insects fed 3 different diets yielded 652,598,015 reads, with raw reads per library ranging from 57.8 to 80.3 million reads, with a mean of 72.5 million. The percentage of reads remaining after trimming ranged between 95.8 and 97.7%, with the average read length being 99.8 bp. The proportion of reads per sample mapping to the reference transcriptome ranged from 94.6 to 96.5%. The number of uniquely mapped reads per sample ranged from 48.4 to 64.2 million, with a mean of 58.8 million. The percentage of uniquely mapped reads ranged from 81.6 to 87.3%. All statistics stated are reported in Supplementary Table 2.

### Diet induced transcriptomic responses in *T. ni* midgut

**Figure 5:**
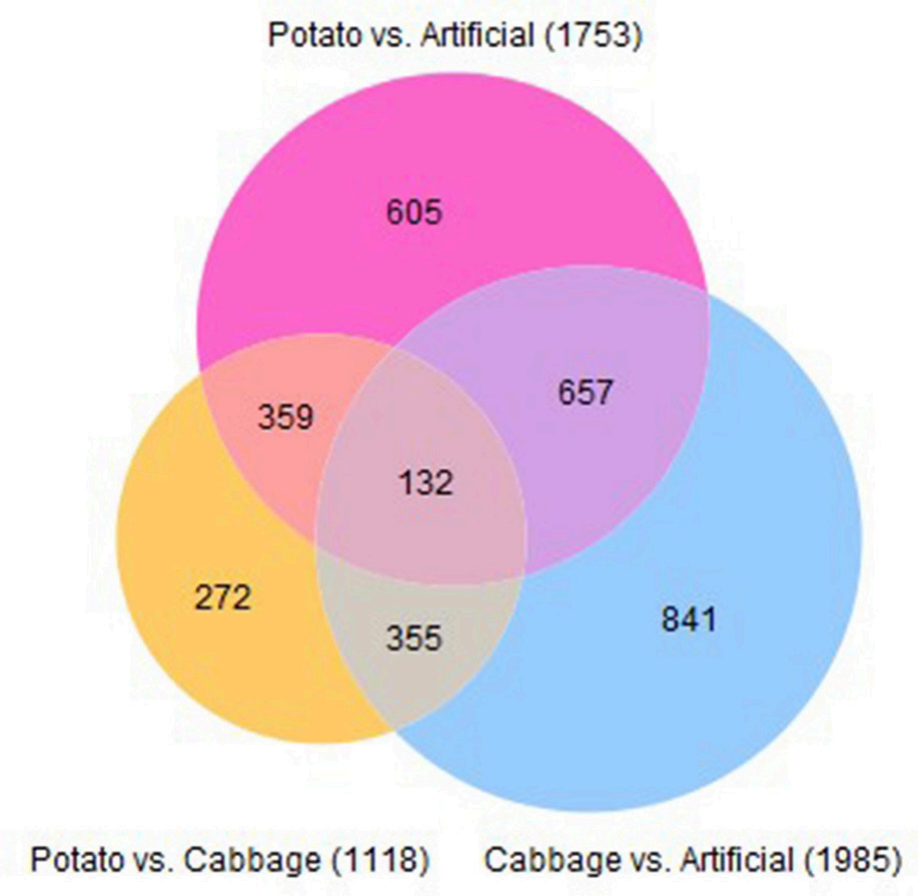
Venn diagram describing the number of contigs significantly differentially expressed in midguts of 4th instar *T. ni* raised on three diets.

Using a cut-off of ≥3 absolute fold change and a FDR-corrected *p*-value of ≤0.001, the total number of contigs significantly differentially expressed for cabbage vs. artificial diet was 1,985; potato vs. cabbage was 1,118 and potato vs. artificial diet was 1,753. There were 132 shared contigs between all diets (Figure 5).

From the contigs that were differentially expressed, we selected those within gene categories encoding products likely to be involved in the PM's architecture, synthesis, and function, for further analysis. The following gene categories were included: structural (peritrophins, mucins, glycoproteins, lipases), microvesicle delivery (gelsolin, annexin), framework (chitin synthase, chitinase, chitin deacetylase) and hormonal effectors (ecdysone and juvenile hormone related). The overall midgut comparisons between 4th instar *T. ni* raised on different diets are presented in Table 1 while the comparison with expanded statistics are given in Supplementary Tables 3–5. Of all the gene categories of interest, the downregulation of contigs corresponding to chitinase and chitin deacetylase were most correlated with diet toxicity.

**Table 1:** Summary of differentially expressed contigs in midguts between all diet group pairs for 4th instar *T. ni*.

**Gene category**	**Contig ID**	**Contig length (bp)**	**Sequence description**	**Cabbage relative to artificial**	**Potato relative to cabbage**	**Potato relative to artificial**
Chitin Synthase	gi|687040850	227	Chitin synthase A	–	–	↓ 13x
Chitinases	11990	1,399	Chitinase domain	↓ 3x	–	–
	17052/16243/ gi|687056067	1,186	Viral-like chitinase	↓ 10x	–	↓ 13x
	17700	676	Chitinase	–	–	↓ 8x
	gi|687027891	9,581	Chitinase 10	–	↓ 4x	↓ 10x
	gi|687027960	3,179	Chitinase 3	↓ 9x	–	↓ 13x
	gi|687094659/8386	1,202	Chitinase 2	↓ 17x	↓ 4x	↓ 68x
	gi|687131816	2,030	Endochitinase A1-like	–	–	↓ 3x
Chitin Deacetylase	16131/11096	1,843	Chitin deacetylase 1	–	↓ 2x	↓ 6x
Glycoprotein	838	4,146	Glycoprotein 1-like	↓ 3x	–	–
	9115	789	Glycoprotein 2B-like	↑ 3x	–	–
	12335	654	Glycoprotein 2B-like	↑ 3x	–	–
	12706	1,400	Glycoprotein 1-like	↓ 4x	–	↓ 4x
	13031	1,431	Glycoprotein 1-like	↑ 3x	–	–
	13710	2,774	Glycoprotein 2C-like	↑ 25x	–	↑ 34x
	13711	2,440	Glycoprotein 2C-like	↑ 32x	–	↑ 42x
	13714	559	Glycoprotein 2C-like	↑ 28x	–	–
	17683	603	Glycoprotein 2C-like	–	–	↑ 6x
	18285	471	Endocuticle structural glycoprotein ABD-5-like	↑ 7x	–	↑ 7x
	18333	590	Glycoprotein ABD-5-like	↑ 8x	–	–
Ecdysone Inducibles	3024	1,783	Ecdysone-induced protein 74EF isoform A	↑ 4x	–	↑ 4x
	5946	453	Ecdysone receptor transcript variant X2	↓ 5x	–	↓ 4x
	6995	571	Ecdysteroid-induced (E75)	–	–	↓ 4x
	8772	1,940	Zinc finger protein on ecdysone puffs	↓ 3x	–	–
	16286	1,185	3-dehydrecdysone 3b-reductase	↓ 4x	–	↓ 6x
	18136	2,470	20-hydroxy-ecdysone receptor	↓ 3x	↑ 15x	↑ 4x
	gi|687100743	380	Ecdysone oxidase gene	↓ 4x	–	↓ 4x
Juvenile hormone inducibles	2789	3,733	Juvenile hormone-inducible protein	↓ 5x	–	–
	4247	1,993	Juvenile hormone epoxide hydrolase precursor	–	↑ 3x	–
	6007	1,020	Juvenile hormone epoxide hydrolase-like	–	↓ 7x	↓ 11x
	9577	2,010	Juvenile hormone esterase-like isoform	↓ 4x	–	–
	11982	494	Juvenile hormone esterase precursor (JHE)	↓ 19x	–	↓ 27x
	13294	2,389	Juvenile hormone epoxide hydrolase-like protein	–	↓ 5x	–
	14365	2,322	Juvenile hormone esterase-like	–	–	↓ 5,811x
	16406	2,351	Juvenile hormone sensitive hemolymph protein	–	–	↓ 8x
	16491	850	Juvenile hormone-inducible protein	–	↓ 3x	↓ 4x
	17075	1,195	Juvenile hormone esterase-like	–	–	↓ 5x
	17228	1,428	Juvenile hormone epoxide hydrolase-like	–	–	↓ 25x
	18269	714	Juvenile hormone binding-like protein	↓ 24x	–	↓ 78x
	gi|687052097	205	Juvenile hormone esterase precursor (JHE)	↓ 25x	–	↓ 35x
Gelsolin	14408	1,099	Gelsolin-like	↑ 3x	–	–
Mucins	2899	4,725	Mucin-12-like	–	–	↓ 8x
	8946	4,324	Mucin-2-like	↓ 3x	–	↓ 6x
	16236	442	Intestinal mucin	–	↑ 10x	↑ 11x
	17180	417	Mucin-5AC	–	–	↓ 7x
	gi|687050021	288	Mucin-5AC	↓ 13x	–	–
	gi|687060229	294	Mucin-5AC-like	↓ 16x	–	↓ 19x
	gi|687064266	219	Mucin-5AC-like	↓ 19x	–	↓ 13x
	gi|687073777	537	Mucin-5AC-like	↓ 17x	–	↓ 10x
	gi|687079129	295	Mucin-5AC-like	↓ 8x	–	↓ 10x
	gi|687080496	259	Mucin-5AC-like	↓ 12x	–	↓ 5x
	gi|687085871	401	Mucin-2-like	↑ 5x	–	–
	gi|687093538	984	Mucin-5AC	–	–	↓ 12x
Lipases	3030	1,018	Lipase 3-like	↑ 8x	↓ 7x	–
	10385	437	Lipase (Lipn004)	–	–	↓ 3x
	10606	1,230	Lipase 3-like	↓ 4x	↓ 6x	↓ 26x
	11520	893	Lipase-like	–	–	↓ 3x
	15809	1,398	Lipase 1-like	–	↓ 4x	↓ 4x
	16200	153	Insect intestinal lipase 7	–	↑ 4x	–
	16325	797	Insect intestinal lipase 6	–	↑ 4x	–
	16375	977	Insect intestinal lipase 6	↓ 3x	↓ 4x	↓ 15x
	16377	660	Lipase 3-like	↑ 3x	–	↑ 6x
	16389	885	Lipase-like	–	↓ 159x	↓ 96x
	16607	559	Lipase ABHD12	↓ 5x	–	↓ 5x
	16887	568	Lipase member H-like	↑ 23x	–	↑ 48x
	17002	670	Bile salt-activated lipase-like	↑ 5x	–	↑ 3x
	17081	483	Lipase-like	–	–	↓ 4x
	17396	880	Lipase 3-like	↑ 69x	–	↑ 35x
	gi|687078584	265	Lipase-like	↓ 26x	↑ 608x	↑ 23x
	gi|687085460	404	Lipase-like	↓ 20x	↑ 468x	↑ 23x
	gi|687101661	671	Lipase-related Protein 1-like	↓ 6x	–	↑ 4x
	gi|687116092	1,317	Lipase-like	↓ 4x	–	↑ 4x
	gi|687125502	266	Lipase	↓ 5x	–	–

*Dash, no significant change; ↑, increased; ↓, decreased*.

## Discussion

### Physiological differences in gut pH with different diets

It has been suggested that larval gut pH may play a role in resistance to pathogens and that gut pH can be significantly altered by diet (Keating et al., 1990). However, while our data supported the idea that diet can influence gut pH levels, as observed between diet treatments (Figure 2), there was no evident relationship between these differences and the significant differences in the susceptibility to the virus in *T. ni* larvae reared on different diets. Larvae reared on potato and artificial diet had an alkaline pH in the midgut well within the 9–11 range reported for most lepidopteran larvae (Dow, 1992), while those raised on cabbage had a less alkaline pH, possibly due to the high ascorbic acid content of cabbage. However, despite having a significantly lower gut pH, larvae fed cabbage were not the most susceptible to the virus, having an LD_50_ in between those reared on artificial diet and potato (Figure 1). The lack of effect may be related to the fact that the pH remained above 8, which is sufficiently alkaline to dissolve OBs effectively (Martin and Martin, 1983; Felton and Duffey, 1991).

### Morphological and transcriptional differences in *T. ni* midgut and PM

**Figure 6:**
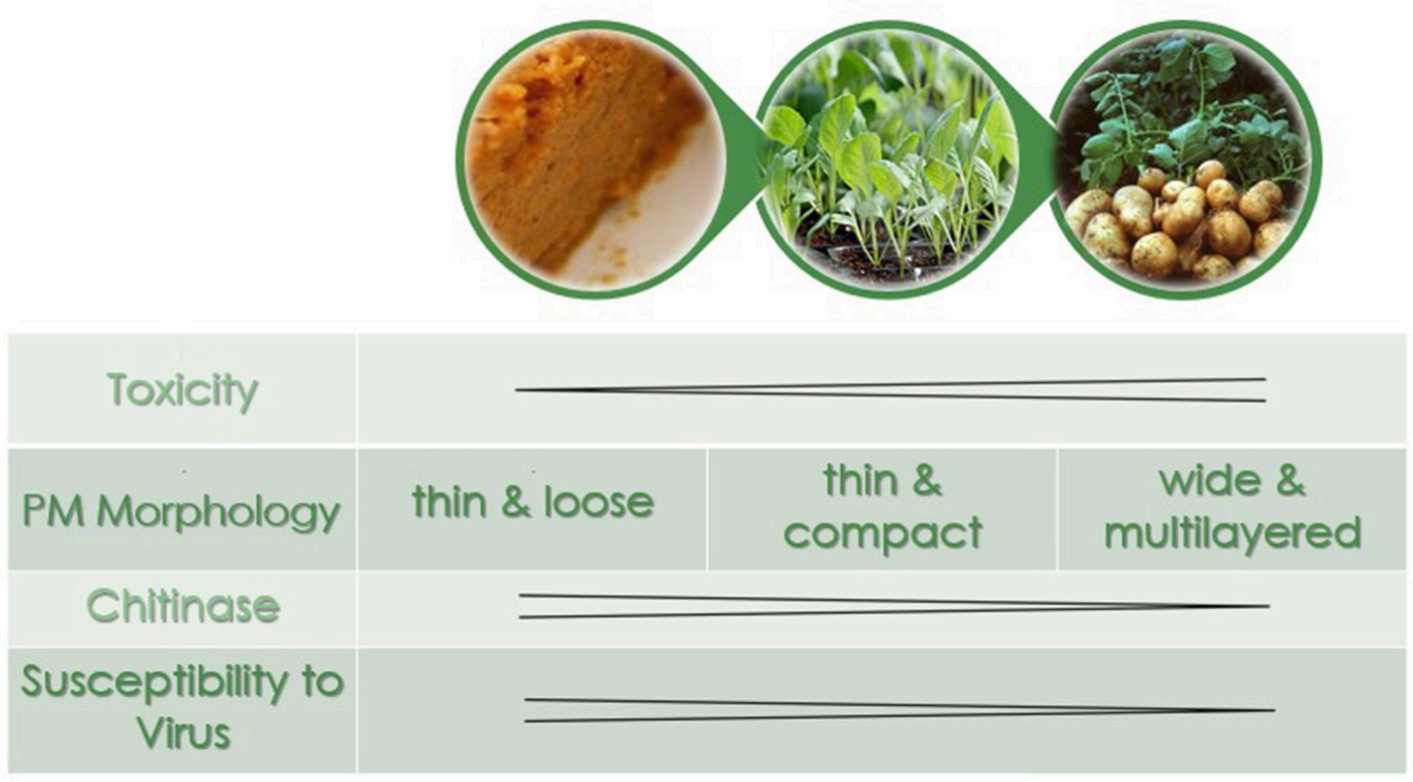
The peritrophic matrix morphology, chitinase expression levels, and susceptibility to virus as a function of diet in 4th instar *T. ni*. In order of increasing toxicity are artificial diet, cabbage, and potato.

Our results suggest that the different susceptibility of larvae reared on different treatments are the result of changes in the PM structure induced by diet, especially in the anterior end of the midgut where most AcMNPV baculovirus infections in *T. ni* occur (Javed et al., 2016). Larvae reared on artificial diet, the most susceptible to the baculovirus, not only had thin (≤1 μm) and fragile PMs, they had high levels of chitinase transcripts (Table 1), the products of which would actively degrade chitin structures like the PM. Thus, such a thin PM would not provide a very effective barrier against viral infection. In comparison, while the PM of larvae reared on cabbage, a preferred host plant, was also thin (≤1 μm), it was more uniform and dense than that seen in larvae fed artificial diet. Furthermore, the lower transcript levels of chitinases observed in the midgut of these insects would result in less degradation of the PM, which could lower the susceptibility to the baculovirus. The LD_50_ for larvae raised on potato, a non-preferred host plant laden with defensive biologically active secondary metabolites (Friedman, 2006), was higher than that for the other two treatments. This could be explained, at least in part by the fact that these larvae had thicker and less organized layers in the PM and the lowest chitinase and chitin deacetylase transcript levels. In addition the PM of larvae reared on potato also had more microvesicles (Figure 4), consistent with repair/reinforcement, as microvesicle membranes become partially soluble in alkaline pH and, when close to the intestinal lumen, release their contents which become incorporated into the PM (Eisemann and Binnington, 1994; Terra, 2001). The proposed relationships between diet chemistry and the corresponding effects on 4th instar *T. ni* PM morphology, chitinase gene expression regulation, and susceptibility to baculovirus are summarized in Figure 6.

Other potential modulators of the PM are the levels of ecdysone- and JH-induced gene expression in the midgut environment, through resulting changes to synthesis of their corresponding protein products. This is because ecdysone and JH work antagonistically to coordinate molting, a process during which the insect's chitin content is drastically altered (Truman and Riddiford, 2002). For example ecdysone induces molting, which is correlated with a thickening of the PM (Toprak et al., 2014), and larvae reared on potato had increased transcript levels of 20-hydroxyecdysone receptor and reduced levels of those for ecdysone oxidase. The latter catalyzes the conversion of ecdysone into 3-dehydroecdysone, diverting it from being processed into the active form, 20-hydroxyecdysone, which acts on ecdysone receptors to stimulate molting (Takeuchi et al., 2005). Thus differential expression of both gene groups could result in a PM with morphological characteristics of a molting, rather than a feeding, larva.

### Detoxification gene responses to plant allelochemicals

The midgut's ability to detoxify plant toxins is an essential characteristic that allows insects, especially generalist species, to cope with a diversity of secondary plant compounds. Many cytochrome P450s are important enzymes in the functionalization step of detoxification (Stevens et al., 2000; Cianfrogna et al., 2002; Li et al., 2002). Glutathione S-transferases (GSTs) convert lipophilic xenobiotics into hydrophilic compounds for excretion or sequestration (Francis et al., 2005; Després et al., 2007) while UDP-glucosyl transferases (UGTs) detoxify benzoxazinoids by conjugation with a sugar (Wouters et al., 2014). All three major detoxifying enzyme families were represented in the top 50 contigs with the biggest changes in midgut transcript levels between larvae reared on potatoes and cabbage (Supplementary Table 6), a pattern that was much more pronounced than when comparing the midgut results of larvae reared on artificial diet with either of the host plants (Supplementary Tables 7, 8). These detoxifying enzyme genes were predominantly downregulated in midguts of potato-raised larvae. A similar response was found in a comparison of midgut transcriptomes of *T. ni* larvae fed on tomato (*Solanaceae*) or *Arabidopsis thaliana* (*Brassicaceae*) (Herde and Howe, 2014), two different plant species from the same families we used in this study. Herde and Howe (2014) hypothesized that anti-nutritive proteins found in *Solanaceae* elicit large-scale remodeling of digestive enzymes and that the metabolic costs associated with digestive flexibility constrain an insect's ability to detoxify secondary metabolites when feeding on plants using this defensive strategy. However, it is difficult to isolate the relative importance of such effects on insect health from those resulting from the thickened PM, as a thicker PM could also reduce digestion and result in decreased nutrition (Plymale et al., 2008), but in nature both probably play a role.

## Conclusions

We found that diet affects the susceptibility of *T. ni* larvae to AcMNPV virus infection and this would appear to be mediated through alterations to the structure of the PM and not through changes to midgut pH. Examination of gene expression in the midgut using RNA-seq showed many changes in gene expression in larvae raised on potato compared with those raised on cabbage or artificial diet, most noticeably with respect to lowered transcript levels of chitinase and chitin deacetylase genes. This could explain the thickened and multi-layered PM seen in these larvae, which could provide a more effective barrier to baculovirus infection. These changes in gene expression provide leads for further experiments, using directed approaches such as RNA interference to selectively silence individual candidate genes, to identify the specific mechanisms involved.

## Author contributions

EC and CD: conceived and designed the experiments; EC, DK, and MO: performed the experiments; MO and ME: contributed reagents, materials, and analysis tools; EC, CD, and JM: wrote the manuscript; DK and ME: edited the manuscript. All authors read and approved the final document.

### Conflict of interest statement

The authors declare that the research was conducted in the absence of any commercial or financial relationships that could be construed as a potential conflict of interest.

## Supplementary material

NCBI Sequence Read Archive submissions can be found under accession SRP124718.
